# Identification of Novel RNA-Protein Contact in Complex of Ribosomal Protein S7 and 3’-Terminal Fragment of 16S rRNA in E. coli

**Published:** 2012

**Authors:** A.V. Golovin, G.A. Khayrullina, B. Kraal, А.М. Kopylov

**Affiliations:** Department of Bioengineering and Bioinformatics, Lomonosov Moscow State University, Leninskie Gory, 1/73, Moscow, Russia, 119991; Chemistry Department, Lomonosov Moscow State University, Moscow, Leninskie Gory, 1/3, Moscow, Russia, 119992; Leiden Institute of Chemistry, Leiden University, 2300 RA Leiden, the Netherlands

**Keywords:** ribosome, initiation, self-assembly, ribosomal protein S7, UV– induced cross-linking

## Abstract

For prokaryotes in vitro, 16S rRNA and 20 ribosomal proteins are capable of hierarchical self- assembly yielding a 30S ribosomal subunit. The self-assembly is initiated by interactions between 16S rRNA and three key ribosomal proteins: S4, S8, and S7. These proteins also have a regulatory function in the translation of their polycistronic operons recognizing a specific region of mRNA. Therefore, studying the RNA–protein interactions within binary complexes is obligatory for understanding ribosome biogenesis. The non-conventional RNA–protein contact within the binary complex of recombinant ribosomal protein S7 and its 16S rRNA binding site (236 nucleotides) was identified. UV–induced RNA–protein cross-links revealed that S7 cross-links to nucleotide U1321 of 16S rRNA. The careful consideration of the published RNA– protein cross-links for protein S7 within the 30S subunit and their correlation with the X-ray data for the 30S subunit have been performed. The RNA – protein cross–link within the binary complex identified in this study is not the same as the previously found cross-links for a subunit both in a solution, and in acrystal. The structure of the binary RNA–protein complex formed at the initial steps of self-assembly of the small subunit appears to be rearranged during the formation of the final structure of the subunit.

## INTRODUCTION 

The *in vitro* self-assembly of bacterial ribosomes has been relatively well described [1–5]. The phenomenology of the events resulting in the formation of individual ribosomal subunits has been well established. However, the thorough analysis of the interaction between rRNA and proteins is just being started. 

The individual assembly of the small 30S ribosomal subunit and the large 50S ribosomal subunit occurs during the formation of the prokaryotic 70S ribosomes. The small ribosomal subunit of *Escherichia coli * consists of 1542-nucleotides-long 16S rRNA and 20 different medium-size proteins. The proteins that are the first to bind to the 16S rRNA (S4, S7, S8, S15) resulting in the formation of the so-called “structural core” of a small subunit play a crucial role during the self-assembly of the 30S subunit [6, 7]. The ribosomal assembly process has come into the focus of researchers again now that the structure of the small subunit of thermophilic and mesophilic ribosomes has been identified using X-ray diffraction (XRD) analysis [8–10]. The possibility of describing the sequence of events during the self-assembly using a specific structural terminology has become real [3, 4, 11]. Moreover, the potential opportunity for interfering in the ribosome biogenesis process may stimulate the designing of fundamentally different and powerful antibacterial agents. 

**Fig. 1:**
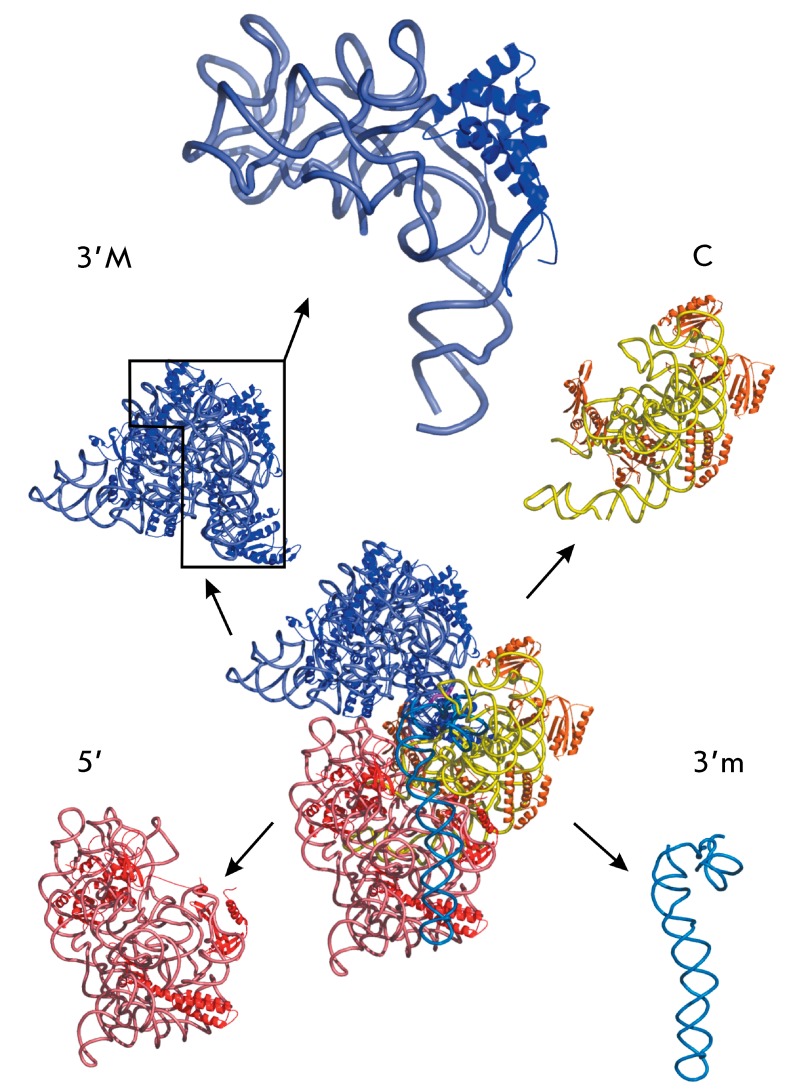
X-ray structure of the 30S small ribosomal subunit isolated from *T. thermophilus* (PDB 1FJF [8]). Domains are specified as follows: (5’) – 5’-end, (С) – central, (3’M) – major 3’-end, and 3’m – minor 3’-end domains. Proteins are shown as dark blue, orange, and red ribbons. 16S rRNA is shown as light blue, cyan, yellow, and pink ribbons. Top: scaled-up 16S RNA (Eco16S)-ribosomal protein S7 complex extracted *in silico*

As opposed to the 50S subunit, the expressive discrete character of the structure of the 30S subunit makes the experimental study of its self-assembly much easier: it consists of 4 domains ( *Fig. 1* ) [8, 9]. Three RNP domains are capable of assembling independently [12–17]. The minimal fragment of 16S rRNA (236 nucleotides long) isolated from *E. coli* (D3LH, Eco16S), which is capable of specific binding to protein S7, the key participant in the subunit assembly process, has been found for the major 3’-terminal domain, [18]. 

The present work is devoted to the investigation of the rRNA–protein contacts in the binary complex of the recombinant ribosomal protein S7 with a binding site on a fragment of 16S rRNA isolated from *E. coli * using UV-induced RNA-protein cross-linking. A non-conventional rRNA-protein contact has been identified: protein S7 is cross-linked to the nucleotide U1321. The annotation of the previously published rRNA–protein cross-links of protein S7 in the 30S subunit in solution and the XRD data obtained for a small subunit crystal has been carried out. The newly identified rRNA–protein cross-link in the binary complex does not match any of the annotated cross-links found in the intact subunit. It can be hypothesized that the structure of the binary rRNA–protein complex that is formed during the initial stages of the small ribosomal subunit assembly must undergo rearrangement during the formation of an intact subunit. 

## EXPERIMENTAL 

T4 polynucleotide kinase (PNK) and PNK buffer (New England Biolabs, USA), reverse transcriptase of the avian myeloblastosis virus (RT-AMV), Taq DNA polymerase, RNase inhibitor, proteinase K, nucleoside triphosphate and its dideoxy derivatives (Roche, Germany), [γ- ^32^ Р]АТР (Amersham, Germany), bovine serum albumin (BSА, MBI, Fermentas, Lithuania), 0.45 µm nitrocellulose filters (Millipore HA, USA;Schleicher & Schuell BA85, Germany), Ni-NTA-agarose (QIAGEN, Germany), phenylmethylsulfonyl fluoride (PMSF, Merk, Germany) were used. The рFD3LH plasmid was kindly provided by L. Brakier-Gingras (University of Montreal, Canada). 

Buffer A: 50 mM Tris-HCl (рН 9.5), 1.5 mM MgCl _2_ , 20 mM (NH _4_ ) _2_ SO _4_ , 1 mM dithiothreitol (DTT), 0.005% NP-40, 5% dimethyl sulfoxide (DMSO), 1 mМ betaine. Buffer B: 40 mM Tris-HCl (рН 7.9), 12 mM MgCl _2_ , 10 mM NaCl, 10 mM DТТ, 2 mM spermidine. Buffer C: 0.3 М NaАс (рН 5.2), 1 mМ EDТА, 0.2% phenol. Buffer D: 50 mM Hepes-KOH (pH 7.0), 100 mM KCl. Buffer E: 50 mM Tris-HCl (рН 8.5), 10 mM MgCl _2_ , 60 mM KCl, 10 mM DТТ, 0.5 mM dNTP. Buffer F: 20 mМ Tris-Ас (рН 7.8); 7 mМ МgAc _2,_ 300 mM NH _4_ Cl, 0.2% BSА. 

**Isolation of the recombinant protein S7 of **

*E.coli* **(EcoS7) and protein S7 of** *Thermus* *thermophilus* **(TthS7) from the superproducer strain of** *E. coli*

The EcoS7 was isolated from the superproducer strain of *E. coli* in accordance with the QIAGEN protocols as was briefly mentioned previously [19]. Cells were collected by centrifugation, suspended in 50mM Tris-HCl (pH 8.0) containing 500 mM NaCl and lysozyme. After the incubation, glycerin was added to 10%; mercaptoethanol, to 5 mM; PMSF, to 0.5 mM; and Triton X-100, to 1%. Following the subsequent ultrasonication, inclusion bodies were dissolved in a buffer containing 8 M urea, applied to Ni-NTA-agarose; after rinsing, the urea concentration in the eluent was reduced to 0. The protein was eluted using a 0–0.5 M gradient of an imidazole solution in a buffer of 50 mM Tris-HCl (pH 7.5), 500 mM NaCl, 1 mM mercaptoethanol, 5% glycerol, and 0.5 mM PMSF. The protein was transferred into the buffer of 20 mM Hepes-KOH (pH 7.5), 100 mM NaCl, 0.2 mM DTT, 5% glycerin, 0.5 mM PMSF by dialysis, and kept at –70 ^0^ С. Prior to complex formation, the protein was transferred into a buffer of 20 mM Tris-HCl (pH 7.6), 4 mM MgAc _2_ , 400 mM NH _4_ Cl, 0.2 mM EDTA, and 4 mM mercaptoethanol. Protein S7 was isolated in a similar fashion [19, 20]. 

**DNA amplification by PCR **

**Fig. 2:**
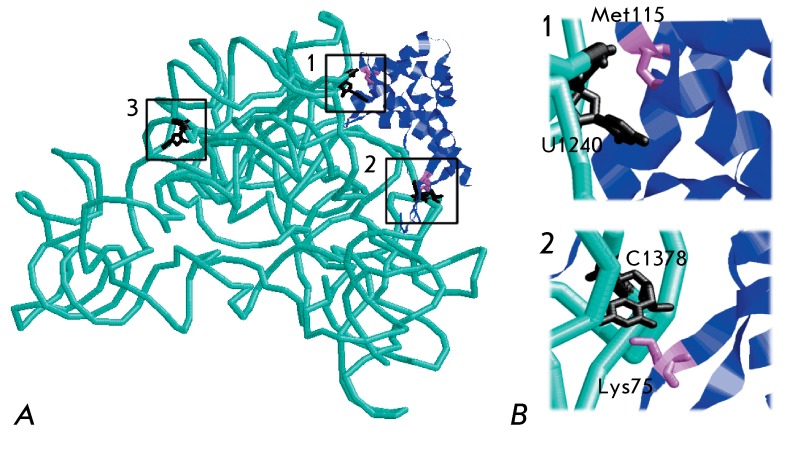
The correlation between the XRD data obtained for the 30S ribosomal subunit isolated from *E.coli* in crystal and data regarding the cross-links of this subunit in solution. A – EcoS7–Eco16S complex structure ( *in silico * extraction from Eco30S). 16S rRNA – cyan ribbon, protein S7– blue ribbon. RNA–protein cross-links are shown in brackets: 1 – U1240-Met115; 2 – C1378-Lys75; 3 - U1321–protein S7 within the binary complex ( *Table* ). B – Details of the RNA–protein contacts are shown in Fig. 2A

**Fig. 3:**
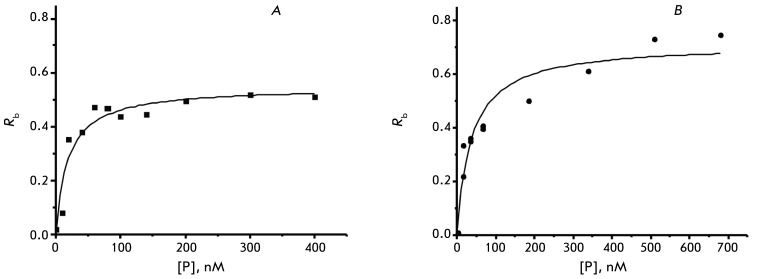
Binding isotherms for the EcoS7–Eco16S complexes (A) and TthS7–Eco16S (B). aK _d_ = 21.5 ± 1.9 nM, and 35.8 ± 9.3 nM, respectively. The initial concentration of the Eco16S – 20 nM, [P] – protein concentration, R _b_ – fraction of the protein bound Eco16S

The matrix DNA fragment was amplified using the рFD3LH plasmid containing cDNA of the minimal fragment of 16S rRNA under the control of the T7 phage promoter. PCR was carried out in 50-400 µl of buffer A containing 200 mM of dNTP, 20 pmol of primers, 50–500 ng of рFD3LH, and 2–5 AU Taq DNA polymerase. The 5’-terminal primer AGGGATCCTAATACGACTCACTATAGGG corresponds to the promoter sequence of the T7 phage RNA-polymerase and is complementary to the vector sequence; the 3’-end primer GTAAGCTTACAAGGCCCGGGAACGTATTCACC is complementary to the fragment G1370-U1393 of the Eco16S (non-complementary sequence is underlined). The primers were synthesized by MWG-Biotech AG company (Germany). PCR was carried out on a thermal cycler (BioRad, USA) under the following conditions: preincubation at –95 ^0^ С for 2 min; cycle at 95 ^0^ С for 45 s; at 60 ^0^ С for 30 s; at 72 ^0^ С for 30–60 s. After 25 cycles, additional incubation at 72 ^0^ С for 7 min was carried out. DNA was purified through electrophoresis in 1–2% agarose gel, 3 volume extraction (according to gel weight) with 6 M NaI (56 ^0^ С, 5 min) with subsequent purification using the PCR Purification Kit (Roche, Germany). 

**Transcription of the 16S rRNA (Eco16S) segment **

**Table:** Analysis of the correlation between the XRD data obtained for the 30S ribosomal subunit of *E. coli* and the data for the cross-links between the 16S rRNA and protein S7 within the 30S subunit in solution

№	Cross-link with the 16S rRNA	Cross-link with protein S7	Distance in the Eco30S, Å	Reagent	Size of the reagent, Å	Reference
1.1	A1238–U1240	S7	3.0	API	8.6	[21]
1.2	A1238–U1240	S7	3.0	IT	5	[22]
1.3*	U1240	M115**	2.7	IT	5	[23–25]
1.4	U1240***	S7	2.7	UV	0	[26]
1.5	16S rRNA	М115**	2.7	UV	0	[27]
2.1	A1377–C1378	S7	3.8	IT	5	[22]
2.2	C1378	K75	3.8	IT	5	[25]

Numeration in the first column: the first number denotes the contact number, 1 (1238–1240) or 2 (1377–1378), the second number is the order number of the cross-link: 1–5 for the first contact, 1–2 for the second contact. API – Methyl- *p* -azidophenylacetimidate; IT – 2-iminothiolane.

* Analogous cross-link was identified in the small subunit of *Bacillus stearothermophilus* (Met115 Bst7) [24, 27].

** In studies [23-25, 27], Met115 was denoted as Met114 (an error in sequencing of protein EcoS7 [28] (R91 was missing [29])).

*** Until 1979, incorrect numeration of the 16S rRNA [30] was used (U1239 instead of U1240).

A) 30S subunit of *E. coli* . 1. Identified cross-link C1265 between the 16S rRNA and protein S7 [30]. C1265 is located at a distance of 35 Å from the nearest amino acid residue of protein S7 in the Eco30S. 2. Identified cross-links 278-280, 1139-1144, 1155-1158, 1531-1542 between the 16S rRNA and protein S7 [31]. The minimal distance between the 1531-1542 segment of the 16S rRNA and protein S7 in the Eco30S is equal to 11 Å. B) The 30S subunit of *B. stearothermophilus:* identified cross-link between the 16S rRNA with the Lys8 residues of protein S7 [27].

*in vitro*

Transcription of Eco16S was carried out on a PCR-copy of matrix DNA containing the T7 phage RNA-polymerase promoter in 100 µl of a solution containing the following components: 2.5 mM NTP, 1000 AU T7 phage RNA polymerase, 60 AU RNase inhibitor, 1 µg/ml of pyrophosphatase, and 4 µg of matrix DNA in buffer B at 37 ^0^ С for 1 h. After the transcription, the solution was subjected to phenol deproteinization followed by chloroform extraction and ethanol precipitation. The RNA was purified in 8% polyacrylamide gel containing 7 M urea and eluted from the gel by diffusion in buffer B. After the elution, the RNA was treated with phenol, chloroform, and subsequently precipitated in ethanol. The precipitated RNA was dissolved in 50 µl of water; the RNA integrity was confirmed using 8% polyacrylamide (containing 7 M urea) gel electrophoresis. The RNA concentration was determined from the absorbance at 260 nm: 1 mg of RNA – 22 o.u. 

**Obtaining the EcoS7–Eco16S and the TthS7–Eco16S complexes**

Complex formation was performed in 200 µl of buffer F. The RNA and the protein were renatured separately at 37°С for 30 min then mixed and heated at 37°С for 30 min. The degree of complex formation was determined using adsorption on nitrocellulose membranes at the filtration rate of 0.5 ml/min by titrating the constant quantity of the ^32^ Р-labeled RNA with an increasing quantity of the protein [19]. The radioactivity of the filters was determined in 10 ml of water in accordance with Cherenkov’s method using a Tracor Analytic counter (France). The apparent dissociation constant (a *K*_d_ ) was determined using XMGRACE software, GNU (http://plasma-gate.weizmann.ac.il/Grace/), by the following equation: 
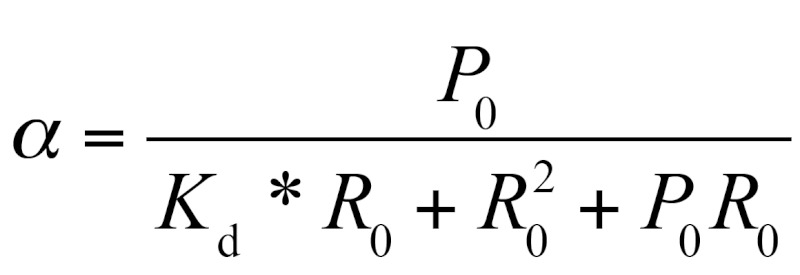


**Fig. 4:**
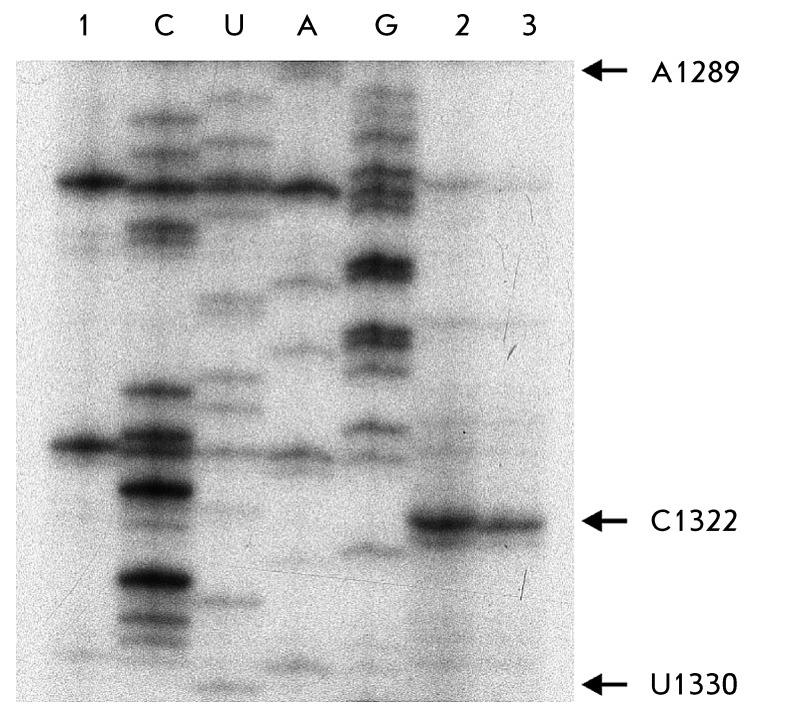
Primer extension analysis of EcoS7-Eco16S, and TthS7 - Eco16S cross-links within the binary complexes. Radioautography of electrophoresis of reverse transcription products in 8% PAAG 8M urea. Lane *1* – cDNA from Eco16S rRNA after UV irradiation. Lanes C, U, A, G – Eco16S rRNA sequencing of the region A1289 – U1330. Lanes 2 and 3 – UV-induced cross-links of Eco16S – EcoS7 and Eco16S-TthS7, respectively. The arrow shows C1322 corresponding to the U1322 cross-link

where *P*_0_ is concentration of protein S7, *R*_o _ is the fixed concentration of Eco16S, *K*_d_ * (a *K*_d_ ) is the apparent dissociation constant, α is the bound fraction in Eco16S complex. 

**UV-induced covalent RNA-protein cross-linking in the EcoS7–Eco16S and the TthS7–Eco16S complexes **

Complex formation was performed in 200 µl of buffer F at the RNA concentration of 150 nM and a 10-fold molar excess of protein. The protein was renatured at 37°С, mixed with RNA, and kept at 37°С for 30 min. The complex was kept under UV light at 260 nm (Stratolinker, USA, power of 2400 µV) on ice for 10 min. The UV light source was located 15 cm away from the complex; the light intensity was controlled by measuring the uridine concentration. 

**Obtaining the oligodeoxyribonucleotide primers labeled with ^32^ P at the 5’-terminus **

The labeled primer (3’-terminal primer for PCR) for reverse transcription was obtained using kination with PNK in the presence of [γ- ^32^ Р]АТР. PNK buffer (10µl) containing 20 pmol of the primer, 3 µl of the [γ- ^32^ Р]АТР (0.4 MBq/µl), and10 AU PNR, and subsequently incubated at 37 ^0^ С for 1 h. The reaction was halted by adding 90 µl of 0.3 M NaAc (pH 5.2) with subsequent phenol deproteinization and chloroform extraction. The primer was precipitated in ethanol and dissolved in 40 µl of water. 

**Mapping the Eco16S nucleotide cross-linked to protein S7 in the EcoS7–Eco16S and the TthS7–Eco16 complexes **

After the irradiation, the complex was treated with proteinase K to remove protein S7. Mapping of the Eco16S nucleotide cross-linked to protein S7 was carried out by reverse transcription using the primer labeled at its 5’-terminus. The hybridization of the primer with RNA was carried out in 4.5 µl of buffer D containing 2–5 pmol of RNA and 0.5 pmol of primer. RNA was denatured at 95 ^0^ С for 1 min followed by slow cooling to 42.5 ^0^ С. Reverse transcription was carried out in 8.5 µl of buffer E containing 2.2 AU RT-AMV at the same temperature for 1 h. One of the ddNTPs (70–400 µM) was added during the control sequencing. Samples were analyzed using 8% polyacrylamide gel containing 7 M urea. 

## RESULTS AND DISCUSSION 

The spatial structures of the 30S small ribosomal subunits isolated from the thermophilic bacterium *T. thermophilus * (Tth30S) [8, 9] and from *E. coli * [10] were determined using XRD. No biochemical data describing the assembly of the 30S subunit in *T. Thermophilus* in solutions have been obtained thus far; only the possibility of domain assembly has been identified [14, 15, 17]. Most biochemical data on the assembly of ribosomes were obtained using *E. coli* ribosomes. Hence, the analysis of the correlation of the biochemical data obtained for the Eco30S in solution and those obtained using XRD for the Eco30S and Tth30S is of particular interest. 

The RNA–protein cross-links were widely used to investigate the contacts in the 30S bacterial ribosome subunit in solution. Several cross-links of the 16S rRNA and protein S7 in the structure of the 30S subunit isolated from *E. coli* ( *Table* ) have been described. Two of these typical cross-links have been reliably identified as U1240–Met115 and C1378–Lys75; this finding correlates well with the XRD data for crystals ( *Table* , *Fig. 2* ). Hence, we used UV-induced cross-linking in the present work to identify the possible rRNA-protein contacts in the Eco16S fragment–protein S7 binary complex. 

It had been previously shown that complexes of protein S7 and the intact 16S rRNA create a cross-link under UV irradiation [32]; however, no cross-linked residues have been identified. 

**Fig. 5:**
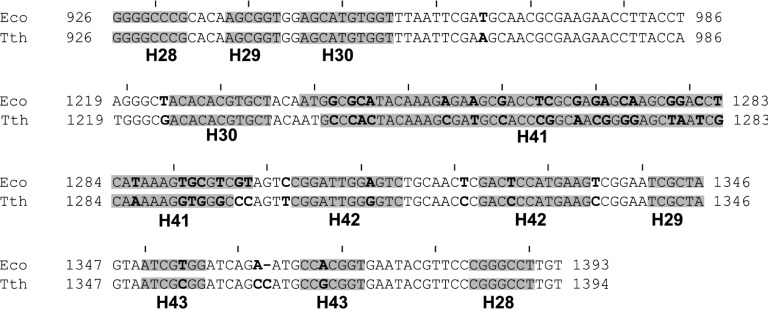
Alignment of the primary structures of the Eco16S and Tth16S fragments. Conventional numbering of nucleotides in the Eco16S was used; the numbering for the Tth16S fragment was in accordance with the PDB 1FJF [12] for Tth30S. Non-identical nucleotides are shown in bold; double-stranded regions are shown in gray

Brakier-Gingras *et al.* have demonstrated [18] that protein EcoS7 is capable of binding to a small fragment of 16s rRNA (236 nucleotides, D3LH, Eco16S), which is the key element in the structure of the major 3’-terminal domain of 16S rRNA. The EcoS7–Eco16S complex was obtained by the authors using EcoS7 isolated from an aggregated ribosomal protein in accordance with the standard methodology [33]. The apparent dissociation constant of this protein complex (a *K*_d_ ) was relatively high (620 ± 80 nM) [18]. The recombinant protein containing 6 additional histidine residues (6 His) at the N terminus was subsequently used. The recombinant protein also bound to the Eco16S; its a *K*_d_ was considerably less, in the range of 110–210 nM [34, 35]. It is considered that the additional fragment containing 6 His residues does not affect the binding of the protein to 16S rRNA [35]; whereas the difference in the constants reflects the difference in isolation methods. A recombinant protein EcoS7 was used in the present work, which had 6 His residues at its N terminus [19].The EcoS7–Eco16S complex turned out to be more stable than it used to be considered [34, 35]; its a *K*_d_ was 21.5 ± 1.9 nM ( *Fig. 3* ), which attests to its high activity. 

The EcoS7-Eco16S complex was irradiated with UV light; the cross-linking efficiency was determined using polyacrylamide gel electrophoresis under denaturing conditions from the ratio between the radioactivity in the RNP zone and the total radioactivity of rRNA. The duration of the irradiation was selected in such a way as to provide maximum yield of the cross-linked RNP. The position of the cross-linked heterocyclic bases in rRNA was determined to identify the Eco16S–EcoS7 contact using reverse transcription after protein hydrolysis with proteinase K; allowance was made for the fact that reverse transcriptase stops one nucleotide before the modified one. The analysis of the “cross-linked” Eco16S-EcoS7 complex ( *Fig. 4* , lane *2* ) definitively identifies the unique stop-signal corresponding to the C1322 nucleotide (cross-linked to U1321).The additional “stop” signals in the remaining locations have not been identified. The position of the cross-link is shown in the tertiary structure of the 30S ribosomal subunit isolated from *E. coli * ( *Fig. 2* ). 

The identified contact between the Eco16S rRNA and protein EcoS7 differs from all the known contacts, which are formed during the cross-linking of the 16S rRNA with protein S7 in a small subunit of *E. coli* ribosomes in solution ( *Table* ). Moreover, the contact between the Eco16S and protein EcoS7 identified by us does not match the structure of the analogues RNP domain in the structure of the 30S *E. coli* subunit in the crystal ( *Fig. 2* ): the amino acid residue of protein S7 closest to U1321 is located at a distance of 35 Å ( *Table* ). 

The difference identified can be attributed to the fact that during the interaction between protein S7 and the 16S rRNA at the initial stages of ribosome assembly the structure of the assembled binary complex differs from the final structure of the corresponding RNP domain within the subunit. Based on the analysis of the structure of the RNP domain in Eco16S and Tth30S, one can assume that the Eco16S in the binary complex with protein S7 is likely to be characterized by an uncoiled state of four-helix bundles (H30, H41, H42, H43), which are packed side-by-side in the crystal structure of Eco16S and Tth30S [19]. Some additional factors may presumably be required for stabilization of the binary complex in its compact state during the self-assembly of ribosomes (e.g., high local concentration of Mg ions [19] or an interaction with the other proteins in the domain). This hypothesis is in agreement with the existence of an additional special Thx protein in thermophilic ribosomes; this protein is of a particularly strong basic character, and therefore, it can stabilize the compact structure of this RNP domain [8, 9]. 

**Fig. 6:**
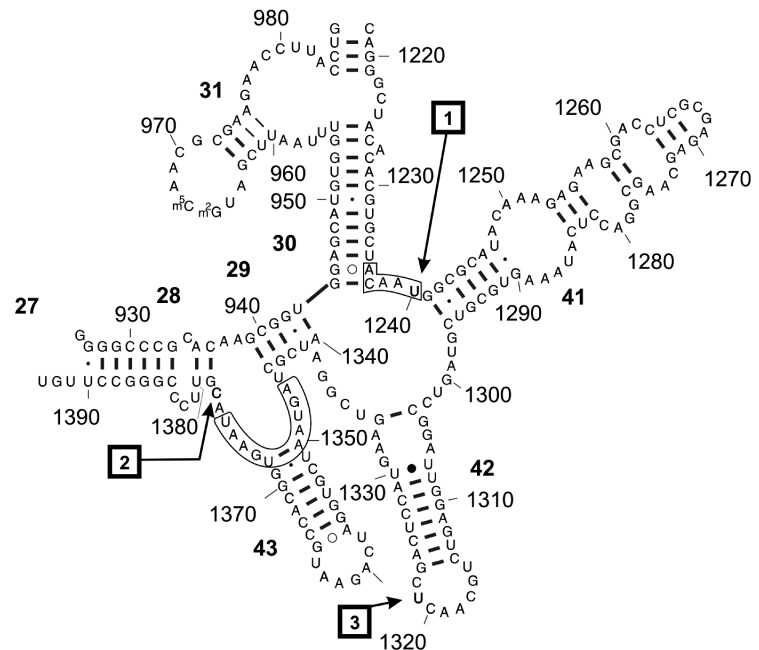
Model of the secondary structure of the major 3’-terminal domain of the 16S rRNA (D3LH, Eco16S) used in this investigation [18]. RNA-protein cross-links are shown by arrows. Cross-links are taken from the *Table* . Cross-links for the 30S *E. coli* subunit: (1) U1240 - M115, (2) C1378 - K75; and for the binary complex: (3) U1321 – protein S7 identified in this work. 16S rRNA sites identical to the streptomycin mRNA binding site for protein S7 are shown in brackets [30]

The comparative analysis of the heterologous structure of the TthS7-Eco16S protein complex is of indisputable interest. In this case, protein TthS7 can be regarded as a “natural mutant” of protein EcoS7 [19]. We had previously shown [19, 36] that ThtS7 can form stable complexes with Eco16S. In the present work, the heterologous complex had a *K*_d _ of 35.8 ± 9.3 nМ ( *Fig. 3* ), which was comparable to the a *K*_d_ of the homologous EcoS7–16S complex (a *K*_d_ = 21.5 ± 1.9 nМ). The contact sites of the recombinant ThtS7 and the Eco16S fragment were also identified in a similar fashion to the Eco16S-EcoS7 complex: protein ThtS7 cross-links to U1321 ( *Fig. 4* ). It appears that a similar RNA–protein contact exists in the heterologous complex. Interestingly, the position 1321 in the 16S rRNA is phylogenetically conserved and the substitution was found only in thermophilic 16S rRNA ( *Fig. 5* ). 

## CONCLUSIONS 

It has been demonstrated in the present study that binary complexes of the ribosomal protein S7 and its local binding site located at 16S rRNA can be obtained for the investigation of the initial stages of the assembly of small bacterial ribosomal subunits. This possibility is in close agreement with the previously shown possibility of assembly of the individual domain RNP complexes of bacterial ribosomes [5, 37]. The S7-containing complex cross-links to the residue of the U1321 under UV irradiation of binary complexes (260 nm) both in homologous (EcoS7-Eco16S) and heterologous (TthS7–Eco16S) complexes. As a result of searching for similar structures in the 16S rRNA and mRNA, Saito and Nomura [38] have proposed that the recombinant protein S7 recognizes a specific motif in the 16 rRNA structure, which is located next to the identified cross-link ( *Fig. 6* ). Moreover, the cross-link of protein S7 and the mRNA fragment next to the tentative motif was identified [39]. The combination of these data argues in favor of Saito and Nomuro’s assumption [38] with regard to the possibility of the initial recognition of this RNA motif by protein S7. 

It can be proposed that the formation of the intact small ribosomal subunit results in reorganization of the contacts in the initial binary complex. Such a rearrangement can also be observed in other RNA–protein complexes; for instance, in complexes of tRNA and phenylalanine-tRNA- synthetase [40]. Some interesting rearrangements have also been identified during the dissociation of binary RNA–protein complexes [41]. 

## Abbreviations

XRD: X-ray diffraction analysis
RNP: ribonucleoprotein
EcoS7, TthS7, BstS7: proteins S7 isolated from*E. coli*,*T. thermophilus *and*B. stearothermophilus*, respectively
Tth30S and Eco30S: small ribosomal subunits isolated from*T. thermophilus*and*E. coli*, respectively
